# Dual-Tasking in Multiple Sclerosis – Implications for a Cognitive Screening Instrument

**DOI:** 10.3389/fnhum.2018.00024

**Published:** 2018-01-31

**Authors:** Christian Beste, Moritz Mückschel, Madlen Paucke, Tjalf Ziemssen

**Affiliations:** ^1^Cognitive Neurophysiology, Department of Child and Adolescent Psychiatry, Faculty of Medicine, Technische Universität Dresden, Dresden, Germany; ^2^Multiple Sclerosis Center, Center of Clinical Neuroscience, Department of Neurology, Faculty of Medicine, Technische Universität Dresden, Dresden, Germany

**Keywords:** multiple sclerosis, dual task, executive function, behavior, psychological refractory period

## Abstract

The monitoring of cognitive functions is central to the assessment and consecutive management of multiple sclerosis (MS). Though, especially cognitive processes that are central to everyday behavior like dual-tasking are often neglected. We examined dual-task performance using a psychological-refractory period (PRP) task in *N* = 21 patients and healthy controls and conducted standard neuropsychological tests. In dual-tasking, MS patients committed more erroneous responses when dual-tasking was difficult. In easier conditions, performance of MS patients did not differ to controls. Interestingly, the response times were generally not affected by the difficulty of the dual task, showing that the deficits observed do not reflect simple motor deficits or deficits in information processing speed but point out deficits in executive control functions and response selection in particular. Effect sizes were considerably large with *d*∼0.80 in mild affected patients and the achieved power was above 99%. There are cognitive control and dual tasking deficits in MS that are not attributable to simple motor speed deficits. Scaling of the difficulty of dual-tasking makes the test applied suitable for a wide variety of MS-patients and may complement neuropsychological assessments in clinical care and research setting.

## Introduction

Due to the high prevalence of cognitive dysfunctions of about 40–70% in multiple sclerosis (MS), the clinical assessment of cognitive dysfunctions is central to the characterization of this disease (Chiaravalloti and DeLuca, 2008; Rocca et al., 2015). Processing speed, memory and attention are the most frequently and earliest affected cognitive domains in MS (e.g., Litvan et al., 1988; Beatty et al., 1989; Demaree et al., 1999; Denney and Lynch, 2009; Amato et al., 2010; Denney et al., 2011; Langdon, 2011). Therefore, cognitive assessment in MS in clinical studies and daily practice usually focuses on decrements in these most prominent domains (Benedict et al., 2006; Amato et al., 2013; Rocca et al., 2015). Opposed to this, executive functions are less examined, possibly because executive deficits are less frequently reported (Rocca et al., 2015) or because testing of this domain is assumed to be more time consuming (Amato et al., 2013; Mückschel et al., 2016).

Executive functions refer to a family of mental processes needed, e.g., when you have to concentrate and pay attention as well as to coordinate different actions (Diamond, 2013). A large body of studies has reported impaired executive functions in MS (e.g., Rao et al., 1991; Foong et al., 1997, 1999; Guimarães and Sá, 2012; Amato et al., 2013; Preston et al., 2013; Mitolo et al., 2015). These functions are particularly relevant in everyday activities requiring the coordination of two or more tasks at the same time, such as driving a car, preparing a meal, or working at your desk in an office. Dual tasking deficits have been reported for many neurological conditions, e.g., closed-head injury (Stablum et al., 2000; Dell’Acqua et al., 2001, 2003), Huntington’s disease (Beste et al., 2012; Vaportzis et al., 2015) or Parkinson’s disease (Nieuwhof et al., 2017; Salazar et al., 2017). Such dual-tasking situations and other employment-related factors are increasingly recognized in the treatment of MS (Krause et al., 2013), and require the hierarchical organization and processing of several individual actions (Dippel and Beste, 2015). It has been shown that dual-tasking conditions, where different cognitive processes related to the selection of actions have to be monitored in parallel, are very sensitive to even slight alterations in the functioning of neuronal networks (Beste et al., 2013a). The efficient usage of widely distributed functional networks including frontal, subcortical, parietal, and primary sensory regions are essential for performance in dual-tasking (Dux et al., 2006; Marois et al., 2006; Szameitat et al., 2006; Stelzel et al., 2008; Chmielewski et al., 2014; Yildiz and Beste, 2014; Gohil et al., 2015, 2017; Stock et al., 2017). Since MS may be described as a disease affecting the human “connectome” (Griffa et al., 2013) and functional connectivity between brain areas is critically affected by microstructural lesions especially in white matter structures (Bonzano et al., 2009, 2011; Droby et al., 2016), dual-tasking processes may be of particular relevance and are very sensitive to detect early and subtle cognitive (executive) dysfunctions in MS. Additionally, it has been shown that dual-task performance is modulated by neurobiological systems (Schulz et al., 2012; Beste et al., 2013b; Yildiz et al., 2013, 2014; Stock et al., 2014) that are either directly or indirectly affected in MS, like the dopaminergic system (Pacheco et al., 2014; Dobryakova et al., 2015). All these facts suggest that dual-tasking abilities are sensitive to key pathophysiological processes in MS. Until now, research on dual-tasking in MS has mainly focused on relatively simple cognitive tasks that are performed during motor activity like walking (Hamilton et al., 2009; Holtzer et al., 2014a,b; Wajda and Sosnoff, 2015; Downer et al., 2016; Learmonth et al., 2017) or balancing (Monticone et al., 2014; Butchard-MacDonald et al., 2017). Studies on dual-tasking during walking consistently showed a slowing of gait in dependence of disease severity in MS (for review, see Leone et al., 2015). Only few studies focused on the effects of walking on the cognitive tasks, but the results are inconclusive (Leone et al., 2015). Hamilton et al. (2009) reported cognitive performance decrements only when task difficulty was elevated, whereas Allali et al. (2014) could not find any significant effect. Overall, since tests on dual tasking in MS have focused on cognition-motor interactions up to now (Wajda and Sosnoff, 2015) they may be more specific for motor aspects than probably assessing cognitive deficits. Therefore, current knowledge on dual-tasking in MS is biased by frequent deficits in motor functions. Additionally, these approaches do not allow to finetune the difficulty of the task, i.e., the amount of cognitive load applied. Established cognitive tests may be not demanding enough to detect subtle cognitive deficits, especially during early stages of MS. The brain is able to compensate for pathological changes in cognitive processes up to a certain point, until these compensatory mechanisms finally break down and manifest as clinically relevant deficits, which is known as cognitive reserve (Rocca et al., 2009; Stern, 2009; Barulli and Stern, 2013). The possibility to adjust the cognitive test to the patients’ performance level may therefore allow to examine the extent of dual tasking and response selection deficits in more detail.

To the best of our knowledge dual-tasking functions have never been examined in MS using procedures that allow a parametrical scaling of the difficulty of dual-tasking in order to detect subtle executive control deficits and that also have a well-established theoretical background regarding cognitive control processes. In the current study we investigated dual-task performance in MS patients using a standard psychological-refractory period (PRP) paradigm (Welford, 1952; Pashler, 1994). Changes in dual-tasking are assumed to provide evidence for early executive dysfunctions in MS. Here, two tasks are presented in close succession and participants are asked to respond as quickly as possible to each task. The term PRP was first used by Welford (1952) to describe the finding that responses (RT2) on the stimulus of the second task (S2) are slower or less accurate when this stimulus was presented shortly after another first stimulus (S1) signaling a different reaction (RT1) (= PRP effect). With increasing time (stimulus onset asynchrony, SOA) between the stimuli signaling different reactions, the PRP effects becomes smaller (Pashler, 1984, 1994; Wu and Liu, 2008). It is assumed that the PRP effect is caused by the postponement of S2 processing due to fully engaged capacity-limited mechanisms still processing S1 (Pashler, 1994; Dell’Acqua et al., 2001, 2003). These central mechanisms likely comprise response selection processes (Van Selst and Jolicoeur, 1997; Van Selst et al., 1999; Dell’Acqua et al., 2003). Therefore, the PRP effect may serve as an index of dual-task interferences on response selection processes. On a neuroanatomical level, it has been shown that the PRP effect is mediated via a widely distributed network involving the superior and middle frontal gyrus (Dux et al., 2006; Marois et al., 2006; Szameitat et al., 2006; Stelzel et al., 2008), as well as areas in the parietal cortex (Hesselmann et al., 2011). Since the PRP task hence depends on widely distributed functional networks, and these are strongly affected in MS (Griffa et al., 2013; Droby et al., 2016), the PRP task is likely to show dysfunctions in MS. However, since dual-tasking measures using the PRP are particularly demanding when the SOA between two stimuli requiring a response is small, we hypothesize that especially short SOA condition should reveal deficits in MS patients, compared to controls.

## Materials and Methods

### Patients and Controls

A sample of *N* = 21 patients was included in this study. Additionally, *N* = 21 healthy control subjects with no history of psychiatric or neurological disease were recruited. Detailed clinical and demographical data including standard neuropsychological tests are shown in **Table 1**. Patients underwent standard neuropsychological assessments using the Beck Depression Inventory (BDI), Modified Fatigue Impact Scale (MFIS) and an assessment of alertness, information processing speed, working memory, divided auditory and visual attention using the “Test of Attentional Performance” (TAP, version 2.3). Importantly, all MS patients had no visual deficits and no auditory deficits.

**Table 1:** Results of neuropsychological testing and demographical data of the MS patient group and the healthy control group.

	MS patients	Control group	*t*	*p*	*d*
Age	39.76 (12.29)	29.52 (5.89)	3.44	0.001^*^	1.05
Education (years in school)	11.34 (0.97)	11.62 (0.8)	–1.04	0.304	–0.33
Years since diagnosis	13.06 (7.41)	N/A			
Age relative to years since diagnosis	6.09 (3.84)	N/A			
Beck Depression Inventory (BDI)	13.95 (10.44)	7.05 (6.81)	2.54	0.015^*^	0.81
Expanded Disability Status Scale (EDSS)	3.32 (1.78)	N/A			
MS Medication (*n*)					
Copaxone	2	N/A			
Fingolimod	3	N/A			
Tysabri	6	N/A			
Other	7	N/A			
none	3	N/A			
Modified Fatigue Impact Scale (MFIS)	34.15 (23.59)	21.86 (11.89)	2.08	0.044^*^	0.72
Sex (*n*)	16 female/5 male	12 female/9 male			
Type of MS (*n*)					
Relapsing Remitting MS (RRMS)	17	N/A			
Secondary Progressive MS (SPMS)	4	N/A			
Test of Attentional Performance (TAP)					
Alertness median RT	303.1 (89.68)	252.5 (44.53)	–2.32	0.026^*^	–0.73
Intrinsic alertness median (T)	40.24 (8.22)	46.05 (8.09)	–2.31	0.026^*^	–0.75
Phasic arousal median (T)	39.71 (6.10)	44.05 (6.06)	–2.31	0.026^*^	–0.83
Working memory level 3 error (T)	53.57 (5.69)	53.29 (6.41)	0.153	0.879	0.05
Working memory level 3 median (T)	45.43 (9.69)	49.52 (8.79)	–1.44	0.159	–0.46
Divided attention auditory median (T)	36.52 (7.10)	45.50 (8.89)	–3.58	0.001^*^	–1.19
Divided attention visual median (T)	46.05 (8.82)	55.65 (6.88)	–3.88	<0.001^*^	–1.11
Divided attention error (T)	47.24 (8.71)	49.65 (7.18)	–0.965	0.341	–0.26
Divided attention misses (T)	47.48 (8.19)	49.45 (6.17)	–0.868	0.391	–0.28

*The mean and standard deviation (SD) are given, together with the *t*-value, *p-*value and effect size *d*. ^∗^*p* < 0.05.*

All control subjects received financial reimbursement. Each participant gave written informed consent prior to study participation and was treated in accordance with the declaration of Helsinki. The study was approved by the local ethics committee of the Medical Faculty of the TU Dresden.

### Dual Task Experiment

**FIGURE 1:**
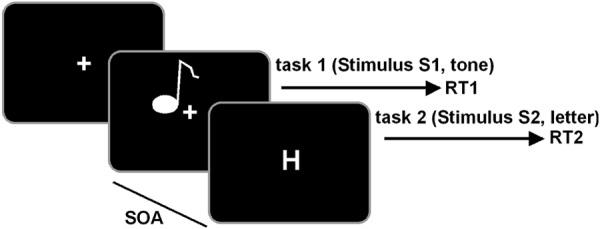
Schematical illustration of the PRP paradigm. The tone task is always presented first and the letter task is always presented second in a defined stimulus-onset asynchrony (SOA). Participants are required to respond first on the tone and second on the letter task.

The task was written in Matlab (The MathWorks Inc.), using the Psychophysics Toolbox. We used a PRP test, comprising an auditory “tone task” and a visual “letter task” (Beste et al., 2013b). The test is well-known to measure cognitive aspects of dual-tasking (Wu and Liu, 2008). The outline of the test is shown in **Figure 1**.

Visual stimuli were presented on a 22” screen at a distance of 60 cm from the participants. Auditory stimuli were presented via headphone. In the tone task, two different sine wave tones were presented, either with a pitch frequency of 300 Hz or 900 Hz. Each tone lasted for 200 ms. In the letter task, white letters, either “H” or “O” (1.8°× 2.3° visual angle), were presented in the middle of the screen. Each trial consisted of the presentation of the tone task (S1), followed by the letter task (S2). The SOA of S2 was predefined to 16, 133, 500, or 1,000 ms. Each SOA was presented in 104 trials, summing up to 416 trials in the whole experiment. The trial sequence was pseudo-randomized. After 208 trials, the participants were allowed to take a short rest between before continuing with the second half of trials. Participants responded to the tone task with their left hand and to the letter task with their right hand. For low tones (300 Hz) participants were asked to press a key with their left middle finger, for high tones the key underlying their left index finger. For the letter task, participants responded with their right index finger for the presentation of an “H” and with their right middle finger for an “O.” Each trial started with the presentation of a central fixation cross, followed by the first stimulus S1 (tone task). The visual S2 stimulus with a predefined SOA was presented for 400 ms and followed by a central fixation cross. The response time window was restricted to 2,000 ms. If no response occurred within this period, the trial was considered a miss. In this case, the next trial started within a randomly jittered interval of 500–2,500 ms (mean 1,500 ms). If a valid response was given, the next trial started after an response stimulus interval (RSI) of 2,000 ms, jittered between 1,000 and 4,000 ms. Participants were asked to respond as quickly and accurately as possible and to place equal emphasis on both tasks. Additionally, the participants were instructed to respond first on the tone stimulus (S1) and second on the letter stimulus (S2). For the analysis of RTs, all trials with response time difference of less than 100 ms between both tasks were removed, to account for possible effects of response grouping. To remove possible RT outliers at the individual subject level, RTs deviating more than 2 SD from the mean where removed.

### Statistics

The data were analyzed in mixed effects ANOVAs with the factor “SOA” as within-subject factor (4-level factor) and the factor “group” (MS patients vs. controls) as between-subject factor (2-level factor). These ANOVAs were run for response parameters (i.e., the relative number of error and reaction times, RTs) for the S1 and S2 stimuli separately. Importantly, responses on the S2 stimuli are the most important one in a PRP paradigm, since only for these responses a response selection bottleneck is evident. Bayes statistics (Wagenmakers, 2007; Masson, 2011) was applied to validate non-significant interaction effects. *Post hoc* tests were Bonferroni-corrected when necessary. Greenhouse–Geisser corrections were applied if necessary. Kolmogorov–Smirnov tests indicated that the data was normal distributed (all *z* < 0.6; *p* > 0.2). Descriptive statistics are provided (mean ± standard error of the mean). Regarding possible effects of age, BDI, MFIS, education (in years of school education) and information processing speed, these factors were controlled for in a separate ANCOVA analysis. Processing speed was estimated using the TAP median response times of the Alertness test. Additionally, RTs and error rates as well as the neuropsychological parameters of the TAP (refer to **Table 1**) were analyzed by means of bivariate Pearson correlation analysis.

## Results

**FIGURE 2:**
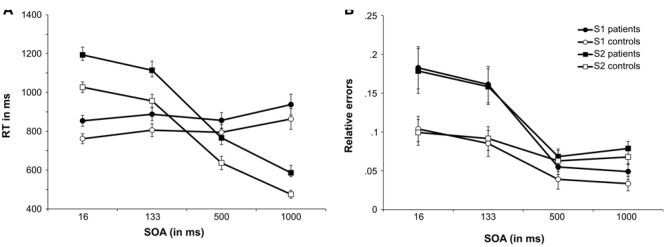
Performance in the PRP task. **(A)** Shows the mean reaction times (RTs) as a function of SOA length for the tone task (S1 stimulus) and the letter task (S2 stimulus) for the MS patients group and controls. **(B)** Shows the mean relative number of errors as a function of SOA length for the tone task (S1 stimulus) and the letter task (S2 stimulus) for the MS patients group and controls. Error bars denote the standard error of the mean (SEM).

The results of the neuropsychological testing as well as demographical and clinical data are summarized in **Table 1**. The behavioral results for response speed and error rates for S1 and S2 responses are shown in **Figure 2**.

### Response Speed

The reaction time data is plotted in **Figure 2A** and accuracy data is plotted in **Figure 2B**. All RTs are given in milliseconds (ms).

The mixed effects ANOVA for S1 RTs revealed a significant main effect of SOA [*F*(1,57) = 8.60; *p* < 0.001; $\eta_{p}^{2}$ = 0.177]. *Post hoc* pairwise comparisons showed that RTs for SOA16 were significantly smaller than SOA133 (*p* = 0.003) and SOA1000 (*p* = 0.007). Additionally, RTs for SOA500 were significantly shorter than for SOA1000 (*p* < 0.001). All other comparisons were not significant (all *p*’s > 0.333). All other main effects and interactions were not significant (all *F*’s < 0.225; all *p*’s > 137). We used Bayesian statistics to validate the lack of group effects on SOA, i.e., to test for the assumption that S1 RTs were not differentially modulated by the factors group and SOA. As proposed by Wagenmakers (2007) the Bayes factor (BF) was estimated using the Bayesian information criterion for the interaction of “SOA × group,” using the sum of squares of the error term and the effect term as provided by the ANOVA. The BF can be converted into the posterior probability that the results are in favor of the null hypothesis (*p*_BIC_(H_0_|D)) by calculating BF/(BF + 1)^10^. The posterior probability in favor of the alternative hypothesis (*p*_BIC_(H1|D)) can be calculated as 1-*p*_BIC_(H_0_|D). For the non-significant interaction of “SOA × group,” the Bayesian analysis showed *p*_BIC_(H_0_|D) = 0.996 and *p*_BIC_(H1|D) = 0.004. This can be considered as very strong evidence in favor of the null hypothesis that group did not modulate SOAs differentially (Raftery, 1995).

However, as outlined above, the response on the S2 stimulus is more important, since the PRP effect becomes evident for responses on the S2 stimuli. For S2, the ANOVA showed that RTs differed between SOAs, as indicated by a significant main effect [*F*(2,94) = 799.68; *p* < 0.001; $\eta_{p}^{2}$ = 0.952]. *Post hoc* pairwise comparisons showed that all SOAs differed significantly from each other (all *p*’s < 0.001). RTs for S2 were longest for SOA16 (1,110 ms ± 24), followed by SOA133 (1,035 ms ± 28), and SOA500 (701 ms ± 29). RTs were fastest for SOA1000 (530 ms ± 21). The significant main effect of group [*F*(1,40) = 7.89; *p* = 0.008; $\eta_{p}^{2}$ = 0.165] showed that patients responded slower (915 ms ± 35) than controls (774 ms ± 35). The interaction “SOA × group” was not significant [*F*(2,94) = 1.74; *p* = 0.174; $\eta_{p}^{2}$ = 0.042]. Bayesian statistics was applied to validate the lack of group-dependent effects on SOA. For the non-significant interaction of “SOA × group,” the Bayesian analysis showed *p*_BIC_(H_0_|D) = 0.991 and *p*_BIC_(H1|D) = 0.009. According to Raftery (1995) this can be considered as strong evidence in favor of the null hypothesis, i.e., SOAs were not modulated differentially by group.

To control for modulating effects of depression and fatigue, we repeated the above analyses using the BDI and MFIS scores as covariates in the model. To account for effects of different educational levels, education was included as covariate by means of school education in years. The covariate analysis (ANCOVA) showed that none of these covariates changed the pattern of results for S1 and S2 RT data (all *p* > 0.17). To control for the age differences between MS and control group, age was included as covariate. The results showed that age did not significantly influence the results (all *p* > 0.19). Information processing speed, as estimated by the median RTs of the TAP alertness test, had a significant influence on S1 RTs [*F*(1,39) = 5.04; *p* = 0.031; $\eta_{p}^{2}$ = 0.114] as well as on S2 RTs [*F*(1,39) = 8.17; *p* = 0.007; $\eta_{p}^{2}$ = 0.173]. After inclusion of response speed as covariate, all other effects on S1 RTs were not significant (all *p* > 0.475). For S2 RTs, only the main effect of SOA remained significant [*F*(3,117) = 41.74; *p* < 0.001; $\eta_{p}^{2}$ = 0.517]. The main effect of group showed a trend toward significance [*F*(1,39) = 3.54; *p* = 0.068; $\eta_{p}^{2}$ = 0.083].

### Response Accuracy

The mixed effects ANOVA for S1 error rates (relative errors) revealed a main effect of SOA [*F*(1,58) = 59.88; *p* < 0.001; $\eta_{p}^{2}$ = 0.600]. *Post hoc* pairwise comparisons revealed that the error rates of SOA16 (0.143 ± 0.016), SOA133 (0.123 ± 0.014), SOA500 (0.047 ± 0.008), and SOA1000 (0.041 ± 0.007) differed significantly from each other (all *p*’s < 0.006), except for SOA500 and SOA1000 (*p* > 0.9). Additionally, the main effect of group was significant [*F*(1,40) = 4.95; *p* = 0.03; $\eta_{p}^{2}$ = 0.110], suggesting that MS patients committed more errors (0.112 ± 0.015) than controls (0.065 ± 0.015). Importantly, there was an interaction “SOA × group” [*F*(1,58) = 6.96; *p* = 0.005; $\eta_{p}^{2}$ = 0.148]. To further analyze this interaction, *post hoc t*-tests were calculated, comparing error rates between groups, separately for each SOA. For SOA16, controls committed fewer errors (0.104 ± 0.023) than MS patients [0.183 ± 0.23; *t*(40) = 2.48; *p* = 0.018; *d* = 0.78]. For SOA133, controls committed less errors (0.085 ± 0.02) than patients [0.161 ± 0.02; *t*(40) = 2.63; *p* = 0.012; *d* = 0.83]. There were no significant group differences of error rates for SOA500 [controls: 0.039 ± 0.011; patients: 0.055 ± 0.011; *t*(40) = 0.999; *p* = 0.324; *d* = 0.32] as well as SOA1000 [controls: 0.033 ± 0.01; patients: 0.049 ± 0.01; *t*(40) = 1.13; *p* = 0.267; *d* = 0.36].

The mixed effects ANOVA for S2 response error rates showed a significant main effect of SOA [*F*(1,60) = 24.11; *p* < 0.001; $\eta_{p}^{2}$ = 0.376]. *Post hoc* pairwise comparisons showed that error rates differed significantly (all *p*’s < 0.001) between SOA16 (0.139 ± 0.017) and SOA500 (0.065 ± 0.008), SOA 16 and SOA1000 (0.073 ± 0.007), SOA133 (0.125 ± 0.014) and SOA500 as well as SOA 133 and SOA1000. No differences were found between SOA16 and SOA133 as well as between SOA500 and SOA1000 (all *p*’s > 0.150). As indicated by a significant main effect of group [*F*(1,40) = 97.75; *p* < 0.001; $\eta_{p}^{2}$ = 0.71] patients were overall less accurate (0.121 ± 0.014) than controls (0.08 ± 0.014). Importantly, there was also a significant interaction effect of “SOA × group” [*F*(1,60) = 6.39; *p* = 0.006; $\eta_{p}^{2}$ = 0.138]. *Post hoc t*-tests were calculated to explore this interaction, comparing group error rates for each SOA. Similar to S1 results, patients (0.179 ± 0.024) committed more errors than controls (0.099 ± 0.024) for SOA16 [*t*(40) = 2.38; *p* = 0.022; *d* = 0.75]. For SOA133, patients (0.158 ± 0.02) were also less accurate than controls [0.092 ± 0.02; *t*(40) = 2.42; *p* = 0.020; *d* = 0.77]. Again, no group differences were found for SOA500 [patients: 0.068 ± 0.012; controls: 0.063 ± 0.012; *t*(40) = 0.328; *p* = 0.745; *d* = 0.10] as well as SOA1000 [patients: 0.079 ± 0.01; controls: 0.068 ± 0.01; *t*(40) = 0.387; *p* = 0.420; *d* = 0.26]. A *post hoc* power analysis using G^∗^power on the basis of the obtained effect size in the interaction ($\eta_{p}^{2}$ = 0.138) revealed that the achieved power was above 99%.

To control for effects of depression, fatigue, and education we repeated the above analyses using the BDI and MFIS scores as well as education in years as covariates in the model. The results show that neither depression, nor fatigue, nor education in years changed the pattern of results (all *p* > 0.38). Additionally, age was included as a covariate. The analysis showed no influence of age (all *p* > 0.51). To account for possible modulatory effects of information processing speed, the TAP Alertness median RTs were included as a covariate. Processing speed had a significant influence on S1 error rates [*F*(1,39) = 5.5; *p* = 0.024; $\eta_{p}^{2}$ = 0.124]. Most important, the observed interaction was still significant [*F*(3,117) = 3.535; *p* = 0.017; $\eta_{p}^{2}$ = 0.083]. This shows that information processing speed does not solely explain the pattern of results and that the applied test does not simply measure aspects of information processing speed. All other effects were not significant (all *p* > 0.17). For S2 error rates, processing speed was not significant [*F*(1,39) = 3.76; *p* = 0.06; $\eta_{p}^{2}$ = 0.088].

There were also no correlations with parameters of the neuropsychological assessment using the TAP (please refer to **Table 1** for a list of all included parameters; all *r* < 0.2; *p* > 0.3), which shows that PRP paradigms examine processes not yet covered in standard neuropsychological procedures applied to screen cognitive dysfunctions in MS.

## Discussion

We examined dual-tasking performance in patients with MS using a PRP paradigm. The results clearly showed impaired dual tasking in MS. These deficits did not affect the speed of responding, but the ability to respond accurately.

The observed relatively small RT differences on responses to the S1 stimulus reflect a typical pattern observed in the PRP (Wu and Liu, 2008). In contrast to responses on the S2 stimulus, the first response is not subject to limitations in response selection capacities. The finding that MS patients did not respond slower on the S1 stimulus shows that differences between groups were not simply due to motor deficits in MS patients, but due to differences in cognitive response selection processes in MS. Moreover, the findings that differential effects between SOA length and group (MS vs. controls) were not evident for the reaction time data, but only for the accuracy data underline that motor response speed is not affecting the pattern of results. As expected, differences between groups were largest in the most difficult conditions upon responses on the S2 stimulus, where both tasks were presented with only a short gap in between (i.e., 16 ms and 133 ms). In these conditions, MS patients committed more errors than controls. In the other SOA conditions, no group differences were obtained. Based upon this it seems that MS patients have stronger limitations in response selection resources than controls. Until now this has not been shown for MS. However, not only responses on the visual (S2) stimulus were less accurate, but MS patients also committed more errors in response to the auditory (S1) stimulus. This effect, where responses on the S1 stimulus are affected by responses on the S2 stimulus, is known as backward crosstalk effect (Miller, 2006; Janczyk et al., 2014) and shows that action goals in dual-tasking are activated in parallel and not in a step-by-step fashion (Janczyk et al., 2014). Overall, the results show that MS is associated with more limited response selection capacities but does not affect the way how responses are selected. It has been shown that the classical PRP effect is mediated via a widely distributed network involving the superior and middle frontal gyrus (Dux et al., 2006; Marois et al., 2006; Szameitat et al., 2006; Stelzel et al., 2008), as well as areas in the parietal cortex (Hesselmann et al., 2011). Additionally, it has been shown that dual-task performance is modulated by neurobiological systems (Beste et al., 2013b; Yildiz et al., 2013; Stock et al., 2014) that are either directly or indirectly affected in MS, like for example the dopaminergic system (Pacheco et al., 2014; Dobryakova et al., 2015). Therefore, it can be assumed that the changes observed in MS patients are due to a combination of structural neuroanatomical and neurobiological factors.

Importantly, the test applied allows scaling the difficulty of dual-tasking by means of different SOAs. This test may therefore be suitable also for adaptive testing in patients with more severe disease symptoms. This test feature is currently lacking in efforts to use multi- or dual-tasking processes in MS. Standard neuropsychological tests, like the Stroop task and the PASAT may also be considered to measure some facets of response selection. However, these tasks do not test dual-task performance and hence the demands on response selection processes are lower. Current research on dual-tasking in MS focusses on simple cognitive tasks that are performed during motor activity (for a review, see Leone et al., 2015) and thus assess rather cognition-motor interference effects. Existing studies on dual-tasking during walking or balancing strongly operationalize this important executive control functions via pure motor processes that are known to be dysfunctional in MS. Therefore, current knowledge on dual-tasking in MS is biased by the frequent deficits in motor functions and cannot provide accurate insights into the cognitive deficits underlying dual-tasking difficulties *per se*. Due to the task-inherent scaling of the difficulty to perform dual tasks (via the SOAs) the test is most likely applicable to a wide variety of MS-patients; i.e., from the mild to moderate to even strongly affected patients.

Clearly, the moderate sample size as well as the heterogeneity of the MS patients included in this study is a limitation. However, the results are robust, as underlined by the strong effect sizes and the fact that the *post hoc* power analyses revealed a power of above 99%. The robustness of the PRP is further proved by the fact, that the inclusion of processing speed as a covariate did not change the main pattern of results. Information processing speed is one of the most affected cognitive domains in MS (e.g., Rao et al., 1989; Demaree et al., 1999). Since the PRP is assumed to be based on an information processing bottleneck (e.g., Pashler, 1984, 1994), processing speed differences may have an impact. Even though both groups differed significantly in processing speed the covariate analysis showed that the observed interaction effect of SOA and group was stable even after controlling for processing speed differences. The age differences between control group and patient group pose a further limitation of this study. Age effects on dual tasking are manifold and associated not only by changes on a neurobiological level but also in strategy and motivation (Bier et al., 2017). However, and most important, the covariate analysis showed that age had no impact on the observed effects in this study. In comparison to other studies of aging on dual tasking (e.g., Li et al., 2001; Verhaeghen et al., 2003; Verhaeghen, 2011; Beurskens and Bock, 2012; Chmielewski et al., 2014; Bier et al., 2017), the age differences as observed here were rather small. Other factors differing between controls and MS patients (e.g., BDI and MFIS) did also not bias the results of this study, as shown by the covariate analysis controlling for these factors. Future studies should incorporate neurophysiological and/or MRI data to examine the neuropathological mechanisms behind the observed deficits in more detail.

The study shows that, using the applied PRP paradigm, multi-tasking deficits could be detected in patients with early stages of MS. Because of its scalability, the PRP allows an adaptive testing of important cognitive processes. The PRP may readily complement neuropsychological assessments in clinical care as well as in research settings, as the PRP is easy to conduct and does not require expensive computer hardware or gait analysis devices. The PRP paradigm as applied in the current study may 1 day serve as a prototype for an improved standard diagnostic instrument to detect executive control and response selection deficits in MS. Future research should focus on further cognitive parameters that can complement the PRP in the detection of cognitive deficits in MS. The current findings hopefully help to establish a new, more sensitive cognitive diagnostic instrument to be included in the clinical care of MS patients that can be applied on different subpopulations independent of disease severity.

## Author Contributions

CB and TZ conceived the study, analyzed the data, and wrote the paper. MM conceived the study and analyzed the data. MP conceived the study and collected the data.

## Conflict of Interest Statement

The authors declare that the research was conducted in the absence of any commercial or financial relationships that could be construed as a potential conflict of interest.
